# Exploring Patient Satisfaction and Determinants in Outpatient Services: A Cross-Sectional Study

**DOI:** 10.7759/cureus.77345

**Published:** 2025-01-12

**Authors:** Palak Mahajan, Aanchal Anand, Samar Hossain, Mukesh Vishwakarma

**Affiliations:** 1 Quality Assurance, Govind Ballabh Pant Hospital, Sri Vijaya Puram, IND; 2 Community Medicine, Andaman & Nicobar Islands Institute of Medical Sciences, Sri Vijaya Puram, IND

**Keywords:** andaman & nicobar islands, healthcare quality, outpatient services, patient satisfaction, tertiary care hospital

## Abstract

Background

Patient satisfaction is a crucial metric for evaluating the quality of healthcare services, particularly in outpatient settings where first impressions significantly influence overall perceptions of care. In the Andaman and Nicobar Islands, geographic isolation and resource constraints present unique challenges to healthcare delivery, making the exploration of patient satisfaction essential for improving the quality of healthcare services. This study investigates satisfaction levels and their determinants among 1298 outpatients at a tertiary care hospital in the Andaman and Nicobar Islands.

Aim and objective

The primary aim of this study was to assess patient satisfaction levels with outpatient department (OPD) services and to identify the factors influencing their experiences. Specifically, the study evaluated demographic variations in satisfaction and explored service-related factors that contribute to positive or negative perceptions of care.

Materials and methods

A cross-sectional study was conducted involving 1298 participants from both urban and rural areas attending OPD services in a tertiary care hospital. Data were collected using a structured questionnaire that captured socio-demographic details, satisfaction ratings, and qualitative feedback. Descriptive statistics were employed to summarize trends, while chi-square tests were used to identify significant associations between satisfaction levels and independent variables, including gender, residence, and waiting time. A five-point Likert scale measured satisfaction, allowing for a detailed analysis of patient experiences across multiple dimensions.

Conclusion

The study highlights the importance of efficient time management, improved communication, and maintenance of hygiene in enhancing patient satisfaction. Our study revealed that despite high overall satisfaction, areas such as waiting time and communication needed improvement. Interventions targeting these factors can significantly improve the quality of outpatient services in the hospital. Continuous monitoring of and addressing patient grievances can further optimize patient experiences in healthcare delivery, especially in geographically isolated regions of India.

## Introduction

Patient satisfaction is a cornerstone of healthcare quality, reflecting the extent to which medical services meet or exceed patient expectations [1]. In the context of outpatient services, where the majority of healthcare interactions occur, patient satisfaction serves as a critical measure of service delivery, influencing healthcare-seeking behavior, adherence to treatment, and overall health outcomes [2,3]. The unique geographical and demographic characteristics of the Andaman and Nicobar Islands present distinctive challenges and opportunities in delivering healthcare services. This remote archipelago relies heavily on its only tertiary care facility to address diverse health needs across a dispersed population [4]. Understanding patient satisfaction in this setting offers invaluable insights into the effectiveness of service delivery and opportunities for improvement.

This study delves into the determinants of patient satisfaction among outpatients of a tertiary care hospital in the Andaman and Nicobar Islands. By identifying key factors that shape patient perceptions, it aims to provide actionable evidence for enhancing healthcare quality and optimizing patient experiences in this unique and remote region.

## Materials and methods

Study design

This study employed a facility-based cross-sectional design conducted over six months, from January to June 2024. The cross-sectional approach was chosen because it effectively captures a snapshot of patient satisfaction levels and identifies key determinants influencing their experiences during a specific time frame.

Study area

The study site chosen was the only tertiary care hospital in Sri Vijaya Puram, which serves as the only referral center for all three districts of the Andaman and Nicobar Islands, making it an ideal setting to evaluate the effectiveness and accessibility of outpatient department (OPD) services.

Study population

Patients attending OPD of GB Pant Hospital, Sri Vijaya Puram.

Inclusion criteria

1. Patients aged 18 years and above who attended any OPD specialty during the study period. 2. Patients who visited the OPD at least twice before in the past six months apart from the date of interview.

Exclusion criteria

1. Patients who were critically ill or unable to participate due to medical conditions. 2. Patients with cognitive impairments or communication barriers that hindered accurate data collection.

Sample size

This sample size was determined using Cochran’s formula for cross-sectional studies, assuming a 50% satisfaction prevalence, a 5% margin of error, and a 95% confidence level [5,6]. An additional 10% was added to account for non-responses, ensuring robust and reliable data. $^{}N=(Z^{2}(1-&alpha;/2)*P(1-P))/d^{2}$, where N denotes the sample size, Z(1 - α/2) represents the value of the standard normal variate corresponding to the level of significance α (which is set at 0.5), d refers to the specified precision on either side of the mean, and P represents the satisfaction prevalence.

Sampling method

Simple random sampling was used to select participants from the OPD attendance registry across all specialties. Patients were assigned unique identification numbers and a computer-generated random number table was used to ensure unbiased selection.

Data collection

The questionnaire was administered by the investigators of the study themselves who are trained healthcare professionals with a specialization in public health. Consent was obtained after the patients completed their consultation with the healthcare providers but before they exited the OPD. This timing ensured that participants were fully aware of the study’s objectives and that their decision to participate did not interfere with their clinical visit. The questionnaire was administered at the exit point of the patient flow after they had completed all required interactions within the OPD ensuring that participants could provide feedback on their entire experience, including waiting time and consultations. A designated area near the exit was allocated for this purpose, ensuring minimal disruption to the patient's schedules. The process was quick and respectful of the participants’ time, taking no more than 20-25 minutes per patient. The questionnaire was divided into three key sections:

*Socio-Demographic Information* 

Questions covered participant age, gender, education, occupation, income, residence, and family structure.

*Satisfaction Level* 

Satisfaction was assessed using a five-point Likert scale ranging from "Very Unsatisfied" (1) to "Very Satisfied" (5). The scale measured various dimensions, including waiting times, staff courtesy, availability of resources, and the clarity of medical advice provided.

Service-Related Factors

Additional questions probed specific aspects of satisfaction with OPD services such as the ease of navigation within the hospital, hygiene standards, and perceived staff competency.

Statistical analysis

The data collected was entered into Microsoft Excel (Microsoft Corporation, Redmond, Washington, USA) and analyzed using SPSS version 27 (IBM SPSS Statistics, Armonk, NY). Descriptive statistics, such as frequencies and percentages, summarized demographic characteristics and satisfaction trends. To simplify the analysis and enhance the interpretability of the data, the five-point Likert scale was converted into a three-point Likert scale. The original scale included the following categories: 'Very Unsatisfied,' 'Unsatisfied,' 'Neither Satisfied nor Unsatisfied,' 'Satisfied,' and 'Very Satisfied. For the purposes of this study, the categories "Very Unsatisfied" and "Unsatisfied" were merged into a single category labeled "Unsatisfied" while "Very Satisfied" and "Satisfied" were combined into a single category labeled " Satisfied". The "Neither satisfied nor unsatisfied" category was retained as a distinct midpoint. The binary responses were left unchanged and analyzed directly. For categorical variables, Chi-square tests assessed associations between independent variables (e.g., gender, residence, and waiting time) and satisfaction levels. The threshold for statistical significance was set at p<0.05, a standard in healthcare research.

Ethical considerations

Ethical approval was obtained from the Institutional Ethics Committee of Andaman & Nicobar Islands Institute of Medical Sciences (ANIIMS). All participants provided informed consent before participation. The study adhered to the ethical principles outlined in the Declaration of Helsinki, ensuring participant privacy and data confidentiality throughout the research process.

## Results

Socio-demographic characteristics

The majority of the respondents in our study were male (69.1%) and aged between 31-60 years (58.6%). Most participants preferred English (89.9%) as their communication language and a significant portion (84%) had an Ayushman Bharat Health Account (ABHA) ID. Satisfaction levels were highest for the doctor’s knowledge (90.6%) and behavior (90.1%), while support staff behavior also received positive feedback (71.1% satisfied). However, waiting time showed relatively lower satisfaction with only 49.8% being satisfied. Additionally, services such as hygiene (82.7% satisfied), availability of medicines (74.7% satisfied), and lab investigations (78% satisfied) were generally well-received (Table 1).

**Table 1:** Distribution of patient characteristics and satisfaction levels with outpatient services (N=1298) Data is presented in the form of frequency (n) and percentage (%). ABHA: Ayushman Bharat Health Account

Variable	Categories	Frequency (n)	Percentage (%)
Gender	Male	897	69.1
	Female	401	30.9
Age group	Less than30 years	489	37.6
	31 to 60 years	760	58.6
	More than 60 years	49	3.8
Language preference	English	1167	89.9
	Hindi	131	10.1
Has ABHA ID	No	208	16
	Yes	1090	84
Doctor's knowledge	Unsatisfied	48	3.7
	Neither satisfied nor dissatisfied	74	5.7
	Satisfied	1176	90.6
Doctor's behavior	Unsatisfied	50	3.9
	Neither satisfied nor dissatisfied	78	6
	Satisfied	1170	90.1
Support staff's behavior	Unsatisfied	125	9.6
	Neither satisfied nor dissatisfied	250	19.3
	Satisfied	923	71.1
Waiting time	Unsatisfied	297	22.9
	Neither satisfied nor dissatisfied	355	27.3
	Satisfied	646	49.8
Overall hygiene and sanitation	Unsatisfied	56	4.3
	Neither satisfied nor dissatisfied	169	13
	Satisfied	1073	82.7
Availability of medicines	Unsatisfied	84	6.5
	Neither satisfied nor dissatisfied	244	18.8
	Satisfied	970	74.7
How would you rate the services regarding lab investigations?	Unsatisfied	78	6
	Neither satisfied nor dissatisfied	208	16
	Satisfied	1012	78
Did the doctor explain everything and address your issues?	No	86	6.6
	Yes	1212	93.4
Did the doctor suggest alternatives for your treatment and involve you in decision-making?	No	107	8.2
	Yes	1191	91.8
Did the doctor provide clear instructions regarding medication and treatment?	No	87	6.7
	Yes	1211	93.3

Satisfaction across age groups

Participants aged 31-60 years, consistently reported the highest satisfaction levels across variables, such as the doctor’s knowledge (58.8% satisfied) and behavior (58.7% satisfied). Younger participants under 30 years displayed relatively higher dissatisfaction rates, particularly with waiting time, where only 35.2% reported satisfaction. Older participants aged above 60 years also reported slightly higher dissatisfaction with aspects like waiting time (19.5%) and availability of medicines (6%). The results indicate significant differences in satisfaction for waiting time (p=0.012), while other variables showed no statistical significance across age groups. Overall, middle-aged participants appeared to have the most favorable perceptions of healthcare services (Table 2). 

**Table 2:** Comparative analysis of patient satisfaction across age brackets (N=1298) ^*^p-value has been calculated using the Chi-square test. ^**^Significant at p<0.05 level. Data is presented in the form of frequency (n) and percentage (%).

Variable	Categories	Less than 30 years		31 to 60 years		More than 60 years		Chi-square (p-value)*
		Frequency(n)	%	Frequency(n)	%	Frequency(n)	%	
Doctor's knowledge	Unsatisfied	12	25	34	70.8	2	4.2	8.02 (0.091)
	Neither satisfied nor dissatisfied	34	45.9	35	47.3	5	6.8	
	Satisfied	443	37.7	691	58.8	42	3.6	
Doctor's behavior	Unsatisfied	14	28	34	68	2	4	4.509 (0.341)
	Neither satisfied nor dissatisfied	35	44.9	39	50	4	5.1	
	Satisfied	440	37.6	687	58.7	43	3.7	
Support staff's behavior	Unsatisfied	56	44.8	62	49.6	7	5.6	5 (0.287)
	Neither satisfied nor dissatisfied	95	38	146	58.4	9	3.6	
	Satisfied	338	36.6	552	59.8	33	3.6	
Waiting time	Unsatisfied	82	27.6	157	52.9	58	19.5	12.89 (0.012)**
	Neither satisfied nor dissatisfied	130	37.0	180	51.3	41	11.7	
	Satisfied	227	35.2	307	47.7	110	17.1	
Overall hygiene and sanitation	Unsatisfied	25	44.6	28	50	3	5.4	5.572 (0.334)
	Neither satisfied nor dissatisfied	69	40.8	91	53.8	9	5.3	
	Satisfied	395	36.8	641	59.7	37	3.4	
Availability of medicines	Unsatisfied	37	44	42	50	5	6	3.105 (0.54)
	Neither satisfied nor dissatisfied	92	37.7	143	58.6	9	3.7	
	Satisfied	360	37.1	575	59.3	35	3.6	
How would you rate the services regarding lab investigations?	Unsatisfied	34	43.6	40	51.3	4	5.1	4.693 (0.320)
	Neither satisfied nor dissatisfied	88	42.3	112	53.8	8	3.8	
	Satisfied	367	36.3	608	60.1	37	3.7	
Did the doctor explain everything and address your issues?	No	31	36	51	59.3	4	4.7	0.250 (0.883)
	Yes	458	37.8	709	58.5	45	3.7	
Did the doctor suggest alternatives for your treatment and involve you in decision-making?	No	46	43	57	53.3	4	3.7	1.142 (0.494)
	Yes	443	37.2	703	59	45	3.8	
Did the doctor provide clear instructions regarding medication and treatment?	No	31	35.6	52	59.8	4	4.6	0.285 (0.867)
	Yes	458	37.8	708	58.5	45	3.7	

Gender-based comparison 

Male participants reported higher satisfaction levels for most variables, including the doctor’s knowledge (91.9% satisfied) and behavior (91.9% satisfied), compared to females, where satisfaction rates were 87.8% and 86.3%, respectively. Statistically significant differences were observed in satisfaction with the doctor’s behavior (p=0.005), waiting time (p=0.037), and overall hygiene and sanitation (p=0.005), where males reported higher satisfaction. However, support staff behavior and availability of medicines received similar ratings from both genders, with no significant differences. Satisfaction with lab services was notably higher among males (81.5%) compared to females (70.1%) (p=0.00). Overall, males demonstrated higher satisfaction levels, particularly in areas requiring doctor-patient interactions and service efficiency (Table 3).

**Table 3:** Gender-specific trends in outpatient satisfaction (N=1298) *p-value has been calculated using the Chi-square test. **Significant at p<0.05 level. Data is presented in the form of frequency (n) and percentage (%).

		Gender				
Variable	Categories	Male		Female		Chi-square (p-value)*
		Frequency(n)	%	Frequency(n)	%	
Doctor's knowledge	Unsatisfied	30	3.30	18	4.50	5.68 ( 0.058)
	Neither satisfied nor dissatisfied	43	4.80	31	7.70	
	Satisfied	824	91.90	352	87.80	
Doctor's behavior	Unsatisfied	31	3.50	19	4.70	10.647 (0.005)**
	Neither satisfied nor dissatisfied	42	4.70	36	9.00	
	Satisfied	824	91.90	346	86.30	
Support staff's behavior	Unsatisfied	82	9.10	43	10.70	0.799 (0.671)
	Neither satisfied nor dissatisfied	174	19.40	76	19.00	
	Satisfied	641	71.50	282	70.30	
Waiting time	Unsatisfied	200	22.30	97	24.20	6.603 (0.037)**
	Neither satisfied nor dissatisfied	230	25.60	125	31.20	
	Satisfied	467	52.10	179	44.60	
Overall hygiene and sanitation	Unsatisfied	35	3.90	21	5.20	10.79 (0.005)**
	Neither satisfied nor dissatisfied	100	11.10	69	17.20	
	Satisfied	762	84.90	311	77.60	
Availability of medicines	Unsatisfied	57	6.40	27	6.70	1.187 (0.553)
	Neither satisfied nor dissatisfied	162	18.10	82	20.40	
	Satisfied	678	75.60	292	72.80	
How would you rate the services regarding lab investigations?	Unsatisfied	45	5.00	33	8.20	21.04 (0.00)**
	Neither satisfied nor dissatisfied	121	13.50	87	21.70	
	Satisfied	731	81.50	281	70.10	
Did the doctor explain everything and address your issues?	No	53	5.90	33	8.20	2.413 (0.120)
	Yes	844	94.10	368	91.80	
Did the doctor suggest alternatives for your treatment and involve you in decision-making?	No	66	7.40	41	10.20	3.011 (0.83)
	Yes	831	92.60	360	89.80	
Did the doctor provide clear instructions regarding medication and treatment?	No	52	5.80	35	8.70	3.807 (0.051)
	Yes	845	94.20	366	91.30	

## Discussion

 A cross-sectional study conducted by Saxena R in 2020 at an Armed Forces tertiary hospital in Northern India reported that 45% of patients were dissatisfied with waiting times, emphasizing the need for targeted interventions to reduce delays [7]. Our study found that 49.8% of participants were dissatisfied with waiting times with Chi-square analysis showing a significant association between waiting time and satisfaction levels (p=0.037). The findings of both the studies align , emphasizing the importance of addressing waiting times to improve satisfaction.

Gupta et al., in their cross-sectional study of doctor-patient relationship conducted in 2023 at a government healthcare facility in New Delhi, India, reported that nearly three-fourths of respondents perceived doctors as competent. Additionally, 70% of participants agreed that doctors respected the dignity of patients and more than half acknowledged that doctors went out of their way to help patients [8]. In our study, over 91% of patients attributed high satisfaction levels to courteous and empathetic interactions with doctors and staff. A significant proportion (90.1%) of participants expressed satisfaction with doctors’ behavior and we observed a strong association between staff behavior and patient satisfaction (p=0.005). These findings underscore the the universal importance of communication and empathy in building trust and improving patient experiences.

In our study, participants aged 31-60 years consistently reported the highest satisfaction levels across various variables, including the doctor’s knowledge (58.8% satisfied) and behavior (58.7% satisfied). Younger participants under 30 years displayed relatively higher dissatisfaction rates, particularly with waiting time, where only 35.2% reported satisfaction. Older participants aged above 60 years also expressed slightly higher dissatisfaction with aspects like waiting time (19.5%) and availability of medicines (6%). Statistically significant differences in satisfaction were observed for waiting time (p=0.012), while other variables showed no significant differences across age groups. Overall, middle-aged participants had the most favorable perceptions of healthcare services. Similarly, Swathi KS et al., in a cross-sectional study conducted in Karnataka in 2023, found that patients aged 31-60 years reported higher satisfaction levels compared to younger and older age groups. [9]. The similarity in the findings highlight a consistent trend of higher satisfaction among middle-aged patients, which may be influenced by factors such as more balanced expectations and a better understanding of healthcare processes.

In our study, male participants consistently reported higher satisfaction levels across most variables, including the doctor’s knowledge (91.9%) and behavior (91.9%) as compared to females whose satisfaction rates were 87.8% and 86.3%, respectively. Statistically significant differences were observed in satisfaction with the doctor’s behavior (p=0.005), waiting time (p=0.037), and overall hygiene and sanitation (p=0.005), where males reported higher satisfaction. Satisfaction with lab services was notably higher among males (81.5%) compared to females (70.1%) (p=0.00). This gender disparity in satisfaction levels is consistent with findings from Singh et al., who noted in a systematic review of studies conducted across India in 2020 that male patients consistently reported higher satisfaction levels due to greater access to healthcare and fewer unmet expectations [10]. Similarly, Rao KD et al. in their cross-sectional study conducted across multiple states in India in 2006 found that male patients reported higher satisfaction levels, attributing the difference to better access to healthcare services and fewer unmet expectations [11]. Sreedhar et al., in a cross-sectional study conducted in 2024 in South India also found a significant gender disparity, with 87.1% of male patients reporting satisfaction compared to 74.4% of female patients (p=0.0005) [12]. This aligns with our findings, reinforcing the notion that gender-specific concerns in healthcare delivery must be addressed. The similarities in the results from these studies, including ours, highlight a consistent trend of higher satisfaction levels among male patients, which can be attributed to factors such as smoother doctor-patient interactions, better healthcare access, and fewer perceived barriers to care delivery.

Strengths

The primary strength of this study lies in its comprehensive approach, which included patients from all specialties of the OPD in a tertiary care hospital, ensuring diverse perspectives on healthcare experiences. The use of a robust sample size of 1298 participants enhances the generalizability of the findings.

Limitations

The study design being a cross-sectional design, only provides a snapshot of patient satisfaction levels. Second, the data relied on self-reported responses which are prone to recall bias and social desirability bias, potentially leading to overestimation of satisfaction levels.

Recommendations

To address the identified gaps and enhance patient satisfaction, several recommendations are proposed. First, the implementation of digital queue management systems can significantly reduce waiting times and improve patient flow. Second, regular training programs for healthcare staff, focusing on communication skills and empathy, are essential to maintaining high levels of satisfaction with staff behavior. Third, targeted efforts are needed to bridge urban-rural disparities, including improving infrastructure and healthcare accessibility in rural areas. Lastly, incorporating patient feedback mechanisms and conducting periodic evaluations of OPD services can help identify and address emerging concerns, ensuring continuous quality improvement in healthcare delivery.

## Conclusions

This study highlights overall patient satisfaction with OPD services at a tertiary care hospital in the Andaman and Nicobar Islands, with over 90% of participants expressing positive experiences. Key determinants of satisfaction included waiting time, staff behavior, and clarity of medical advice, underscoring the importance of operational efficiency and empathetic communication in shaping patient perceptions. Age and gender differences in satisfaction were evident, reflecting the need for targeted interventions to address inequities in healthcare delivery.

While the findings align with similar studies across India, they also reveal unique challenges specific to the remote setting of the islands, such as infrastructural limitations and geographic isolation. Addressing these gaps through digital queue management systems, enhanced training for healthcare staff, and improved rural healthcare facilities can further elevate patient satisfaction levels.

This study emphasizes the critical role of patient-centered care and provides actionable insights for policymakers and healthcare administrators to optimize OPD services. Future research focusing on longitudinal analyses and in-depth qualitative evaluations is recommended to gain a more comprehensive understanding of evolving patient needs and expectations.
